# Fatal occupational injuries in fishing, farming and forestry 2010–2015

**DOI:** 10.1093/occmed/kqae073

**Published:** 2024-08-19

**Authors:** M Nazarihaghighipashaki, B E Moen, M Bråtveit

**Affiliations:** Department of Global Public Health and Primary Care, Centre for International Health, University of Bergen, Bergen, Norway; Department of Global Public Health and Primary Care, Centre for International Health, University of Bergen, Bergen, Norway; Department of Global Public Health and Primary Care, Occupational and Environmental Medicine, University of Bergen, Bergen, Norway

## Abstract

**Background:**

Every year, 2.3 million people worldwide succumb to work-related accidents and illnesses. The primary industries have long been acknowledged with elevated accident risks. Recent levels and trends of injury and associated fatalities in these sectors are uncertain. An enhanced understanding of these risks in these industries is required for effective injury prevention in the future.

**Aims:**

This study aimed to describe registered fatalities in the primary industries worldwide, exploring potential disparities between countries and identifying trends in injury rates.

**Methods:**

Data were obtained on fatal occupational injuries per 100,000 workers in farming, fishing and forestry for the years 2010–2015 from the International Labour Organization ILO-STAT database. Descriptive statistics and mixed-model regression analyses were conducted. Fatal occupational injuries in upper- and middle-income countries were compared.

**Results:**

The study incorporated data from 32 countries: 21 from Europe. America had the highest mean occupational fatality injury rate (76.9). The highest recorded rates for individual countries occurred in Colombia in 2014 (265.2) and Lithuania in 2015 (75.0), and the lowest in Greece in 2012 (0.2). Significant variation in injury rates was evident among the countries. There was no trend in the incidence of fatal injuries from 2010 to 2015, neither for all countries, nor Europe. Middle-income countries had higher occupational fatality injury rate than upper-income countries.

**Conclusions:**

The occupational fatality injury rate exhibited considerable variation, ranging from 0.9 to 265.2, and the injury rate was lowest in upper-income countries. There was no trend in the figures 2010–2015.

## Teaser

The global occupational fatality injury rate within primary industries (farming, fishing and forestry) varied from 0.9 to 265.2 during the period 2010–2015. Upper-income countries had lower injury rates. No significant time trend was observed. The data, from the International Labour Organization ILO-STAT database, included information from 32 countries, but several nations had incomplete data. These findings emphasize the necessity for improvements in both reporting mechanisms and preventive measures for occupational injuries within these industries.

**Key learning points**

**What is already known about this subject:**

- The primary industries, including farming, fishing and forestry, have long been recognized for their heightened risk of fatal accidents.
- Limited studies of accidents and accident prevention in the primary industries have been published.
- Greater insight into rates and trends for accidents in the primary industries would help inform the planning of future preventive actions to reduce the accident rate.

**What this study adds:**

- The present study highlights a high fatality rate in the primary industries across many countries during the period 2010–2015.
- The occupational fatality rate is lower in upper-income countries than in middle-income countries.
- There appears to be no change in the fatality rate in the primary industries over the studied 6-year period, indicating a lack of improvement.
- Many countries have not reported their fatal occupational injuries to the International Labour Organization database.

**What impact this may have on practice or policy:**

- There is a crucial need for global improvement in the health and safety issues of the primary industries, especially in middle-income countries.
- The reporting routines for injuries in many countries need enhancement to ensure the completeness of data in the International Labour Organization database; this is particularly important for continents outside Europe.
- Future studies using data from the primary industries from the International Labour Organization database should be conducted to evaluate and address the current situation.

## Introduction

Occupational injuries contribute to a high number of fatalities every year. According to the International Labour Organization (ILO), there were 380,000 fatal occupational injuries worldwide annually in 2019 [1].

Workers in the primary industries, being agriculture, fishing and forestry, represent large occupational groups with well-known high risks of injuries [2]. Agriculture, employing more than a third of the global workforce, ranks as the world’s second-largest employer after the services sector. Farmers utilize a wide range of machinery, including tractors and harvesters, work with animals prone to biting or kicking, and may be exposed to pesticides leading to poisoning [3].

With over 17 million people engaged in fishing worldwide, ships serve as hazardous workplaces, located at sea and exposed to various weather conditions. Common work injuries in fishing include drowning, gear-related accidents, slips and falls [4].

The forestry sector, employing 33 million workers globally, remains one of the most perilous occupations despite technological advancements, with risks associated with tree felling, the use of chainsaws and the handling of other dangerous equipment [5].

Due to the elevated risks of injuries in these primary industries, substantial efforts have been undertaken to improve safety. across numerous countries [6–9]. However, there is a paucity of studies focusing on these industries, and patterns in the incidence of injuries and fatalities between different countries remain unclear. Whether the number of injuries is rising or in decline, and the extent of variability in incidence between different countries, is unknown. Such information would be valuable in the planning of new safety interventions for workers in the primary industries.

Since its establishment in 1919, the ILO has convened governments, companies and workers from 187 member countries to establish labour standards, formulate laws and implement programmes fostering decent employment for all women and men [10]. ILO has instituted the ILO-STAT database to record important work-related information from countries worldwide. The ILO collects data on occupational injuries across various types of work globally, urging countries to submit their information annually to the ILO [11]. These figures are gathered to create global statistics, essential for the development and evaluation of policies aimed at promoting decent work and reducing accidents in workplaces worldwide. However, there are few studies utilizing this data, and to our knowledge, none with a specific focus on primary industries. Also, the difference in injury figures related to the countries’ income category would be of interest, as one might expect better figures from upper-income countries.

Therefore, the objective of this study is to compile and summarize the registered fatalities in the primary industries worldwide, aiming to discern potential differences between the countries and identify any time trends in injury rates. Our hope is that this study will play a role in raising awareness about the critical need for injury prevention in these high-risk occupations. The findings from this research could potentially benefit workers in farming, fishing and forestry by providing insight into the patterns of fatal occupational injuries in these primary industries.

## Methods

This is a descriptive study, using secondary data from the ILO database ILO-STAT, with no individual names or IDs. The dataset is open for any type of analysis or study. It contains labour statistics from all countries reporting to ILO and has a built-in data explorer. Technical requirements are having the Windows computer operating system and using the Google Chrome Browser [12].

The variables obtained were the occupational injury rate from the primary industries, defined as the number of cases of fatal occupational injuries per 100,000 workers. Most of the registered data from primary industries in the ILO-Stat are aggregated, meaning that the figures from the three types of primary industries (agriculture, fishing and forestry) are merged. Since not all countries have professional fishermen, data from landlocked countries were excluded from the study. We decided to include data on occupational injury fatalities for a 6-year period, before the pandemic coronavirus disease 2019 (COVID-19), since the pandemic may have influenced activities and reporting. As the ILO does not have data for all years for all countries, we decided to include data from countries that had at least once reported injury data in the period chosen: 2010–2015. Before the study started, it was discovered that most data were missing from many countries regarding fishing, farming and forestry industries after 2015. The time span was chosen to have as complete data as possible. Data were obtained from the ILO-STAT database in October 2023. The World Bank country classification was used to categorize the countries into classes according to income level: upper income and middle income [13]. No country in our material was in the low-income category.

The data found in the ILO-STAT database were analysed using the IBM Statistical Package for the Social Sciences (SPSS), version 28.01.0. Descriptive statistics were used to calculate means and percentages. Mixed-model regression analyses were performed to assess potential time trends, with fatal injury rate as the dependent variable, year as fixed factor and country as random factor.

Ethical approval was not sought for this study, since we used secondary data from the ILO database, data with no personal identifiers. In Norway, there is no requirement for ethical approval for studies using secondary data. Also, the present study reports figures of injuries only, not specific diagnoses or any other health information.

## Results

In the ILO-STAT database, 32 countries met the inclusion criteria. Among them, 21 countries had provided registered data from Europe (Table 1). Only two landlocked countries had data in the specified 6-year period. Only one African country had registered data, while Oceania had two countries, and America and Asia had four countries each. The highest occupational fatal injury rates for individual countries were seen in Colombia in 2014 and in Lithuania in 2015, with 265.2 and 75.0, respectively. Conversely, the lowest fatality injury rates were seen in Greece, followed by the Netherlands and Poland.

**Table 1.** Fatal occupational injury rates per 100,000 workers in the primary industries in countries with a maritime coastline and fatalities registered in the ILO-Stat database for the period 2010–2015: the separate years and the mean of the whole period

Continent	Country	2010	2011	2012	2013	2014	2015	2010–2015	Continent
Africa									30.9
	Egypt			24.1		29.6	39.0	30.9	
America									76.9
	Colombia			9.4	11.8	265.2		95.5	
	Guadalupe (Mexico)					58.2		58.2	
	USA			56.9	50.3	64.9	57.7	57.5	
Asia									19.5
	Israel			23.1		17.2	9.2	16.5	
	Malaysia		48	27	25		42	35.5	
	Philippines		9.1		48.7		10.9	22.9	
	Turkey			6.4	2.1	1.9	2.0	3.1	
Europe									11.5
	Belgium				4.6		7.5	6.1	
	Croatia			12.6	10.0		2.6	8.4	
	Cyprus			15.4	8.8			12.1	
	Denmark			17.2	16.1	10.4	4.9	12.2	
	Estonia			6.9		4.2	5.4	5.5	
	Finland			1.0	5.0	5.8	5.7	4.4	
	France	0.8	59.6	1.1	0.4	7.0	9.6	13.1	
	Germany			3.4	2.7	2.9	2.2	2.8	
	Greece			0.2		0.4	4.3	1.6	
	Ireland			30.3	15.1	23.9	19.2	22.1	
	Italy			11.4	11.6	11.6	11.3	11.5	
	Latvia			18.3	20.0	22.5	17.1	19.5	
	Lithuania			21.0	8.3	23.4	75.0	31.9	
	The Netherlands			0.4	1.8	1.4	0.3	1.0	
	Norway	8.1	7.4	10.7	18.3	85.5	28.4	26.4	
	Poland			1.1	0.7	0.6	1.2	0.9	
	Portugal			5.5	6.0	6.4	9.3	6.8	
	Romania			36.0	22.7	27.7	30.6	29.3	
	Spain	2.8	3.9	6.3	6.3	11.5	6.5	6.2	
	Sweden			5.8	3.0	10.0	6.1	6.2	
	Ukraine				11.0	9.5	11.7	11.9	
	United Kingdom	10.9	13.7	12.9	13.0	11.0	10.1	11.9	
Oceania									14.8
	Australia	10.7	17.8	18.6	17.0	13.9	15.6	15.6	
	New Zealand			14.0				14.0	

The mean injury rate for each continent was calculated. A blank space means missing data.

**Figure 1.**
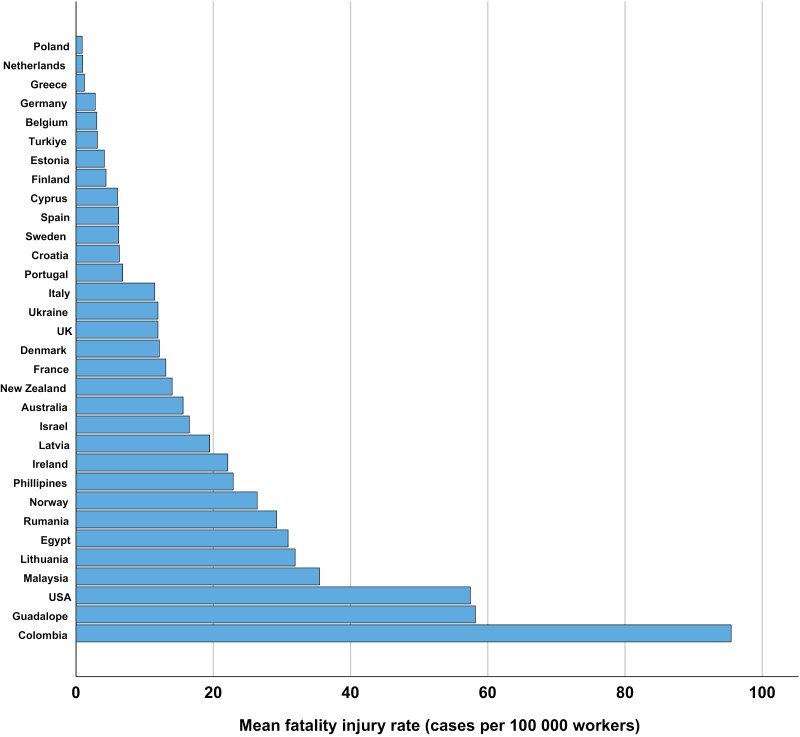
The mean occupational fatality injury rate (cases per 100,000 workers) for primary industries in different countries, registered in the ILO-Stat database 2010–2015.

Australia and Spain were the only countries with registered figures for all 6 years. The figures from Australia were relatively constant throughout the period. The figures from Spain were also quite constant, except for the year 2014, when 11.5 deaths per 100,000 workers were recorded—nearly twice as high as in the other years. Calculating the mean fatal injury rate for each country across the years, 2010–2015, revealed a wide variation among the different countries (Figure 1).

**Figure 2.**
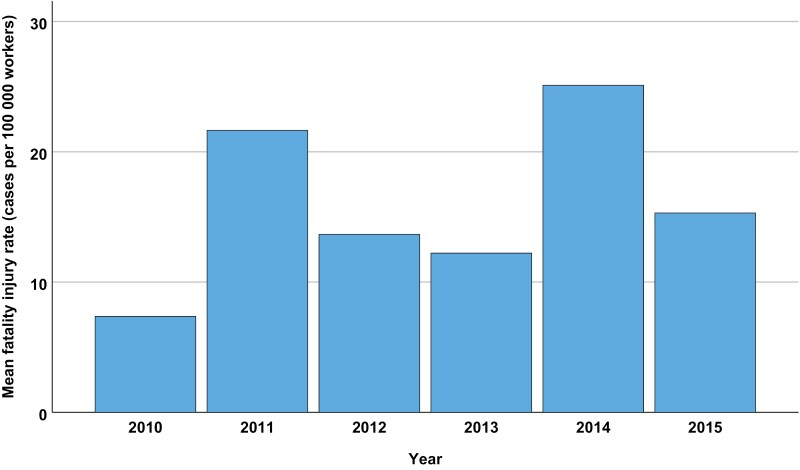
The total annual mean occupational fatality injury rate (cases per 100,000 workers) for primary industries registered in the ILO-Stat database 2010–2015.

**Figure 3.**
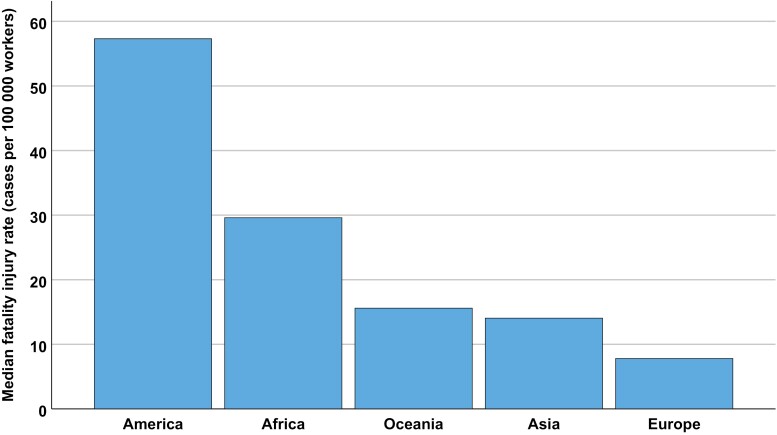
The total annual median occupational fatality injury rate per 100,000 workers for primary industries registered in the ILO-Stat database 2010–2015, shown for each continent.

**Figure 4.**
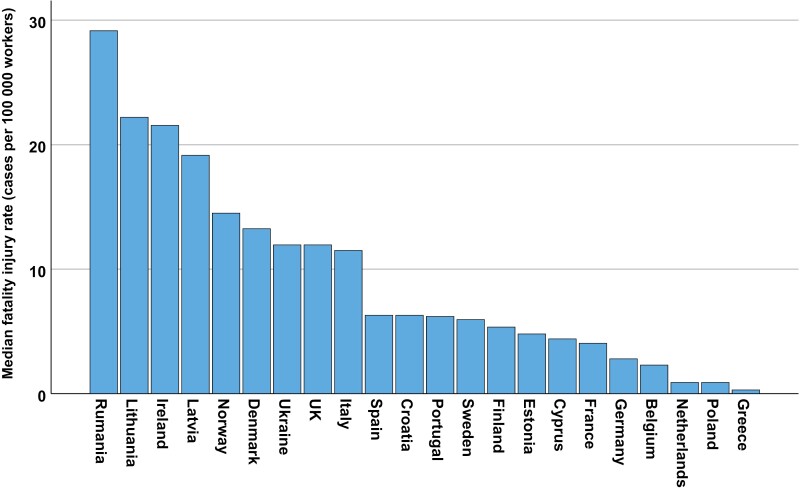
The total annual median occupational fatality injury rate per 100,000 workers for primary industries registered in the ILO-Stat database 2010–2015, shown for each European country.

The highest mean fatal injury rate for all countries occurred in 2014, while the lowest was in 2010 (Figure 2). Among the continents, America had the highest median (median of the means of each year) fatal injury rate, whereas Europe had the lowest (Figure 3). A separate analysis was made for Europe since this continent had data from more countries (Figure 4). Romania, Lithuania, Ireland, Latvia and Norway had the highest median fatality rates (Figure 4).

Six countries were classified to be in the middle-income group (The Philippines, Egypt, Ukraine, Colombia, Guatemala and Malaysia), while all the other countries in our study were in the upper-income group. The fatal occupational injury rate per 100,000 workers was significantly higher in the middle-income group (mean 35.9; standard deviation 56.1; median 18.9), compared to the upper-income group (mean 12.9; standard deviation 15.9; median 8.5; Mann–Whitney *U*-test, *P* < 0.001).

There was no significant time trend in the occupational fatal injury rates over the years 2010–2015 in the mixed-model analyses, neither for the whole material (β = 1.64, *P* > 0.05) nor for European countries separately (β = 0.72, *P* < 0.5).

## Discussion

The global occupational fatality injury rate within primary industries (farming, fishing and forestry) exhibited substantial diversity in the present study, from 0.9 to 265.2. The fatal occupational injury rate per 100,000 workers was significantly higher in the middle-income group, compared to the upper-income group. No significant trend was seen in the figures from 2010 to 2015.

The strength of this study lies in accessing data from a global database, where all countries worldwide are expected to report their fatal occupational injuries to the ILO. This database, designed to provide data for various types of industries, not only the primary sectors, was established independently of the present study, minimizing information bias related to the primary industry under investigation. However, using the ILO database is also a weakness, as the database exhibits incomplete data for certain years and countries. While all countries are urged to report their data to ILO annually, this appears to have been inconsistently executed. The reasons may include inadequate reporting systems for work injuries in the individual countries, insufficient coordination of data gathering and/or inadequacies in submitting data to the ILO. This might cause a bias in our results, but it is difficult to know how this inadequate reporting has influenced the results. Past studies have documented under-reporting of fatal occupational injuries [14], and this might be the situation for some countries. On the other hand, high injury rates might not always mean that the occupational health situation is worse than in other countries, as some countries with a good reporting system and who report to ILO regularly, might have higher figures than the countries who lack good reporting systems.

Another limitation of the present study is that the ILO database aggregates fatality data for fishing, farming and forestry, rather than providing data for each type of primary industry. While this aggregation allows for comparisons across years and countries, it is less useful for developing targeted preventive measures for the distinct work types. Additionally, the study is constrained by its relatively short time frame of 6 years (2010–2015). A more extended observation period might have been preferable, but data for many countries regarding fishing, farming and forestry industries were unavailable after 2015. The selected time frame aimed to maximize data completeness and provide relatively recent information.

The limited number of included countries posed challenges for conducting more sophisticated statistical analyses, and future studies would benefit from including a larger number of countries. Also, we had no information about the size of the public versus informal sector of the workplaces in the countries, and this type of information might have shed more light on the explanations of the different reporting numbers. However, achieving this would necessitate updates of the ILO database.

There are limited prior studies on occupational fatal injuries within the primary industries. A global report on fatal injury rates from 2010 indicates that the rate for agriculture varied from 7.8 in high-income countries to 24.0 in a group of Asian countries [15]. This report employs different country groupings for the continents than the ones we use in our present study, but both studies highlight substantial differences in reported injury rates between the continents. In our present study, European countries, mainly upper-income countries, exhibit lower injury rates compared to Asian countries. However, the 2010 report focuses solely on agriculture and does not include data on fishing and forestry, as presented in our present study. This makes it challenging to compare the actual rates. Additionally, the figures in the 2010 report [15] were obtained through estimations using data from the ILO database and several other data sources, not solely ILO data as in our present study.

The European Union report describes data on fatal accidents at work from 2021 [16], which is 6 years later than our present study. Despite this time gap, the findings in this report align with similar figures in our study, indicating the highest fatality rate in Lithuania and the lowest rates in Poland, Greece and the Netherlands. While the EU report does not provide separate data for the primary industries, it mentions in the text that construction, transportation and storage, manufacturing and agriculture, forestry and fishing sectors together accounted for two-thirds of the total fatal accidents at work that year. This suggests a heightened risk of fatal accidents in the primary industries.

The countries that were categorized as upper-income countries had significantly lower injury rates than the middle-income countries. This might reflect that upper-income countries have resources to focus on health and safety, while resources for health and safety are less available for middle-income countries.

Looking at the results from European countries only, we also see a large variation in the injury rates, and it is difficult to interpret the figures. The working situations must clearly play an important role, but also other cultural and financial issues might be of importance for understanding the differences. It is not obvious why Lithuania has the highest injury rates and Poland has the lowest.

The occupational accident rates of fishery, agriculture, construction, health and social industry in the member states of the European Union have remained notably high for several years [17].

The Bureau of Labor Statistics in the United States reported fatal injury rates for farming, fishing and forestry in the USA for 2020, 2021 and 2022 to be 25.3, 20.0 and 23.5, respectively [18]. While these figures are lower than those reported in our present study, it is important to note that they represent later years. Although they might suggest a potential reduction in fatal injuries within the primary industries in the USA, this cannot be affirmed. The US report also underscores the challenges in gathering injury data, with the process being subject to regular revision.

The present study reveals a consistently high fatality rate in the primary industries across many countries, with no observable change over the 6 years studied, with no trend indicating either a reduction or an increase. Given the elevated figures in comparison to other industries, this underscores a pressing need for improvements in the health and safety practices within the primary industries on a global scale. A critical concern highlighted by the study is the incomplete reporting of fatal occupational injuries to the ILO database by many countries. This is unfortunate, as comprehensive, and accurate data would have been invaluable in devising optimal strategies for global health and safety initiatives. There is a clear need for enhancement in reporting routines, and future studies should be conducted to assess and address this situation.
